# Preclinical Safety Evaluation of a Replication-Defective Canine Adenovirus Type 2 Vector-Based SARS-CoV-2 Vaccine Candidate in Murine Models

**DOI:** 10.3390/vaccines14080647

**Published:** 2026-07-23

**Authors:** Denis Omara, Christian Ndekezi, Susan Mugaba, Angella Nakyanzi, Fortunate Natwijuka, Anne Kapaata, Frank Kato, Drake Byamukama, David E. Ggwaabya, Orla Mugulusi, Freddie Bwanga, David P. Katete, Enock Matovu, Joseph Olobo, Ekii Andrew Obuku, Obondo James Sande, Jennifer Serwanga, Stephen Cose, Pontiano Kaleebu, Sheila N. Balinda

**Affiliations:** 1 Uganda Virus Research Institute (UVRI), Entebbe P.O. Box 49, Uganda; christian.ndekezi@lshtm.ac.uk (C.N.); anakyanzi@uvri.go.ug (A.N.); pontiano.kaleebu@mrcuganda.org (P.K.); sheila.balinda@mrcuganda.org (S.N.B.); 2 Department of Immunology and Molecular Biology, School of Biomedical Sciences, College of Health Sciences, Makerere University, Kampala P.O. Box 7062, Uganda; fortunate.natwijuka@lshtm.ac.uk (F.N.); fxb18@case.edu (F.B.); davidkateete@gmail.com (D.P.K.); joseph.olobo@mak.ac.ug (J.O.); obondo.sande@mak.ac.ug (O.J.S.); 3 Medical Research Council/Uganda Virus Research Institute & London School of Hygiene and Tropical Medicine (MRC/UVRI & LSHTM), Uganda Research Unit, Entebbe P.O. Box 49, Uganda; susanmugaba@my.unt.edu (S.M.); anne.kapaata1@lshtm.ac.uk (A.K.); frank.kato@lshtm.ac.uk (F.K.); dbyamukama@jcrc.org.ug (D.B.); enrine.ggwaabyadavid@students.mak.ac.ug (D.E.G.); orla.mugulusi@lshtm.ac.uk (O.M.); andrew.obuku@lshtm.ac.uk (E.A.O.); jennifer.serwanga@mrcuganda.org (J.S.); stephen.cose@mrcuganda.org (S.C.); 4 Central Laboratory for Animal Research Facility (CLARF), College of Veterinary Medicine, Animal Resources and Biosecurity (COVAB), Makerere University, Kampala P.O. Box 7062, Uganda; enock.matovu@mak.ac.ug

**Keywords:** canine adenovirus type 2 (CAV-2) vector, SARS-CoV-2, vaccines, preclinical study, safety, mouse model

## Abstract

**Background:** Adenoviral vectors are widely used in vaccine development; however, pre-existing immunity to common human adenovirus serotypes can limit their effectiveness. Canine adenovirus type 2 (CAV-2) is a non-human adenovirus with low seroprevalence in humans, making it a suitable alternative vector. Despite its promise, comprehensive preclinical safety data for CAV-2 vector-based vaccine platforms remain limited. In this study, we evaluated the safety profile of a replication-defective CAV-2 vector expressing the Omicron BA.4 SARS-CoV-2 spike immunogen in BALB/c mouse models. **Methods:** The SARS-CoV-2 CAV-2 vector-based vaccine was expressed and propagated in AD293 cells. The mice received intramuscular prime-boost immunisations with low (1 × 10^6^ PFU), moderate (0.5 × 10^10^ PFU), or high (1 × 10^10^ PFU) vaccine doses, alongside empty CAV-2 vector and physiological buffer control groups, and were monitored longitudinally up to Day 72. The mice were clinically assessed at days 0, 7, 21, 42, and 72 for any deviations from normal conditions in comparison to the control groups. Biochemical analyses were performed to evaluate liver and kidney function, as well as any tissue injury due to the vaccine candidate. Hematological parameters were assessed by conducting complete blood counts. Body temperature and weight changes were also monitored as an indicator of systemic toxicity. **Results:** The biochemical and haematological parameters remained within physiological reference ranges across all dose groups and timepoints, with no dose-related deviations, indicating that the vaccine candidate did not show evidence of hepatotoxicity, nephrotoxicity, tissue or haematological toxicity. Body temperatures remained within normal physiological ranges following both prime and booster immunisations, and body weights increased normally across all groups as the animals grew throughout the study period without any abnormal weight gain or loss. **Conclusions:** These results suggest that the replication-defective CAV-2-vectored SARS-CoV-2 vaccine candidate was well tolerated and did not demonstrate evidence of systemic toxicity under the conditions tested. These findings demonstrate that the CAV-2 vector exhibits a favourable safety profile in murine models when used as a vaccine delivery platform, supporting its translational potential as an alternative adenoviral vector-based vaccine platform. Further studies incorporating additional safety endpoints, including histopathological, vector persistence and shedding evaluation, are warranted to support continued development of the platform.

## 1. Introduction

Adenoviral vectors have emerged as one of the most versatile and widely used platforms in vaccine development and gene delivery due to their ability to induce robust humoral and cellular immune responses; they can infect a wide variety of dividing or nondividing cells; they are easily purified to high titres; the strains commonly used to construct recombinant viruses are well characterized; they can accommodate up to 37 kb of foreign genetic material; their genomes can easily be manipulated; their genome rarely integrates into the host chromosome; they are genetically stable and have established manufacturing processes, making them suitable for transient gene expression applications [1,2]. Adenoviruses are generally species-specific, of which the predominantly used ones for vector development include human, chimpanzee, bovine, canine, porcine, ovine, and fowl adenoviruses [3,4,5,6].

For vaccine applications, adenoviral vectors are frequently engineered as replication-defective to improve safety while maintaining efficient antigen delivery and expression [7]. Vector selection depends on factors including the target pathogen, desired immune response, pre-existing anti-vector immunity, and transgene requirements [1]. These features have contributed to the widespread use of adenoviral vectors in modern vaccine development. Traditional human adenovirus serotypes such as Ad5 and chimpanzee adenovirus-derived vectors (e.g., ChAdOx1from Ad25) have been deployed in several successful vaccine candidates, particularly during the COVID-19 pandemic, highlighting the accelerated utility of adenoviral vectors in response to emerging infectious diseases [8]. Adenoviral vector-based vaccines against SARS-CoV-2 induced protective immunity in both preclinical and clinical studies, demonstrating safety and efficacy in humans and underscoring the translational promise of this platform for pandemic preparedness and vaccine development against a broad range of pathogens [8].

Despite these advances, the widespread use of human adenovirus vectors is constrained by the presence of pre-existing anti-vector immunity in human populations, which can diminish vector transduction efficiency, reduce antigen expression, and limit vaccine efficacy [9]. Furthermore, rare adverse events, such as vaccine-induced immune thrombotic thrombocytopenia (VITT), although uncommon, have been associated with some adenoviral vaccines, reinforcing the need for alternative vectors with favourable safety profiles [10]. Non-human adenovirus vectors offer a promising solution to these challenges. Canine adenovirus type 2 (CAV-2) vector represents one such alternative, characterised by low seroprevalence in humans and reduced likelihood of neutralisation by pre-existing immunity [11]. Early studies of CAV-2 vector and its engineered derivatives have demonstrated efficient transgene delivery and neuronal tropism, with growing interest in their application as vaccine platforms and gene therapy vectors [11,12].

Although CAV-2 has been investigated as a vaccine and gene-delivery platform in several preclinical studies, safety data for individual vaccine constructs remain an important component of translational development, particularly where different transgenes, manufacturing processes, dosing regimens, and intended clinical applications are involved. Murine models serve as a foundational step in assessing adverse effects and systemic toxicity under controlled experimental conditions before advancing to higher-order species or clinical trials. Such studies not only inform the safety profile of vaccine candidates but also elucidate potential biomarkers of toxicity and inform dose selection for downstream research. In this study, we report a detailed preclinical safety assessment of a non-replicative CAV-2 vector expressing the spike SARS-CoV-2 antigen as the immunogen in BALB/c mice following intramuscular administration by evaluating clinical observations, body weight and temperature trends, as well as haematological and biochemical parameters. This investigation contributes to the effort to establish the foundational safety information required for the continued development of CAV-2 vector-based vaccine platforms.

## 2. Materials and Methods

### 2.1. Non-Replicative CAV-2 Vector-Based Vaccine Development Against SARS-CoV-2

#### 2.1.1. The Recombinant Non-Replicative CAV-2 Vector Design

The non-replicative CAV-2 vector used in this work was obtained from the Institut de Génétique Moléculaire de Montpellier (IGMM). The CAV-2 vector was engineered by deleting the E1 and E3 genomic regions to render the virus replication-defective and increase its transgene-carrying capacity, respectively. The parental transfer vector contained a cytomegalovirus (CMV) promoter-driven Dre recombinase expression cassette. For construction of the vaccine candidate, the Dre recombinase coding sequence was replaced with the full-length SARS-CoV-2 spike glycoprotein gene derived from the Omicron BA.4 variant, which was placed under the control of the same CMV promoter. Recombinant CAV-2 plasmids were generated using established molecular cloning approaches that have been widely applied in the development of CAV-2–based vectors [11] and subsequently refined through advances in bacterial recombination technologies and vector engineering strategies [13]. The resulting recombinant plasmid encoded the Omicron BA.4 spike immunogen as the sole transgene expressed by the CAV-2 vector.

#### 2.1.2. Expression of Non-Replicative CAV-2 Vector

The CAV-2 vector genome was released from the plasmid using *PacI* restriction enzyme (Cat. R0547S, New England Biolabs, Ipswich, MA, USA). The non-replicative CAV-2 vector was expressed and expanded in the AD293 cell line (Cat 240085, Agilent, Santa Clara, CA, USA). AD-293 cells express adenoviral E1 genes, which are essential for rescuing and propagating E1-deleted adenovirus vectors. Once inside the cells, the linearised viral genome is recognised by the host cell machinery. The virus is replicated, assembled, and released into the culture medium as recombinant adenovirus particles. The vaccine virus stock was harvested and purified using the Vivapure^®^ Adenopack™ 20 kit (Cat. S-AVPQ022, Sartorius, Gloucestershire, UK) based on the ion-exchange liquid chromatography principle. The purified vaccine virus stock was then titered using the Improved Kärber method to determine the median (50%) tissue culture infectious dose (TCID_50_). The resulting titres were converted to plaque-forming unit (PFU) equivalents using a standard TCID_50_-to-PFU conversion factor, and the PFU values were used to calculate the vaccine doses administered in the study.

#### 2.1.3. Vaccine Formulation

Each vaccine shot had a total volume of 50 μL containing a total of either 1 × 10^10^ PFU (high dose), 0.5 × 10^10^ PFU (moderate dose) or 1 × 10^6^ PFU (low dose) of the vaccine in a physiological buffer containing 20 mM of Tris-HCl (Cat. T5941, Sigma-Alrich, Darmstadt, Germany), 25 mM of sodium chloride (Cat. S7653, Darmstadt, Germany) and 2.5% of glycerol (Cat. G5516, Darmstadt, Germany), pH8. The NaCl salt in the buffer acts as the stabiliser [14]. This is recommended for downstream experiments like infection of model animals and tissue cultures. The physiological buffer enables prolonged storage of vaccine in a −80 °C freezer for up to 2 years, at least 3 months at −20 °C and 1 month at 4–8 °C [15]. Neither preservatives nor adjuvants were added since preservatives can affect the vector, and the vector itself provides potent innate immune stimulation and efficient antigen presentation; addition of an adjuvant may induce excessive inflammation, hence there being no need for the adjuvant in this case [15,16]. Consequently, several licensed and experimental adenoviral vector-based vaccines like the ChAdOx1 nCoV-19 vaccine [17] and Ad26.COV2.S COVID-19 Vaccine [18,19] against SARS-CoV-2 have been developed and evaluated without the incorporation of additional adjuvants. The safety evaluation reported in this study was therefore performed using the vaccine formulation as intended for administration. Vaccine doses were selected to span a broad range of vector exposures and to enable evaluation of potential dose-related safety effects, consistent with preclinical development strategies commonly used for adenoviral vector vaccines. Similar approaches employing defined low-to-high adenoviral vector doses have been reported in murine and translational vaccine studies [20,21,22,23].

### 2.2. Preclinical Safety Evaluation of the Vaccine in Murine Models

#### 2.2.1. Study Design

This was a blinded preclinical study where the blinding was achieved by assigning colour codes to the treatment groups instead of using the actual product names or administered doses (Table 1). A total of 75 BALB/c mice, comprising 45 males and 30 females aged 6–7 weeks, were used in the study. Pregnant females were excluded. (Table 1). The sample size was determined based on previous experience with preclinical vaccine safety studies, ethical considerations to minimise animal use, and the requirement to assess multiple safety endpoints across five predefined sampling time points. A formal a priori power calculation was not performed. There were 5 test groups, composed of 3 vaccine test groups (i.e., low dose, moderate dose and high dose) and two control groups (i.e., empty vector and physiological buffer) (Table 1). A mouse was vaccinated intramuscularly (i.m.) into the quadriceps muscle of the hind limb with a total volume of 50 μL of either the vaccine candidate or control substance. The veterinarians who administered the vaccines, monitored the mice and collected the samples were blinded by colour coding both the vaccine and the control test groups (Table 1). There was a total of 15 cages, each carrying 5 mice, and each test group was allocated 3 cages (i.e., 15 mice, including both males and females) (Table 1). To cater for any bias in the study, cage numbers were randomly allocated to the cages, the 75 mice were randomly picked from a pool of mice that all had initially qualified to be included in the study, and the mice were randomly placed into the cages.

**Figure 1:**
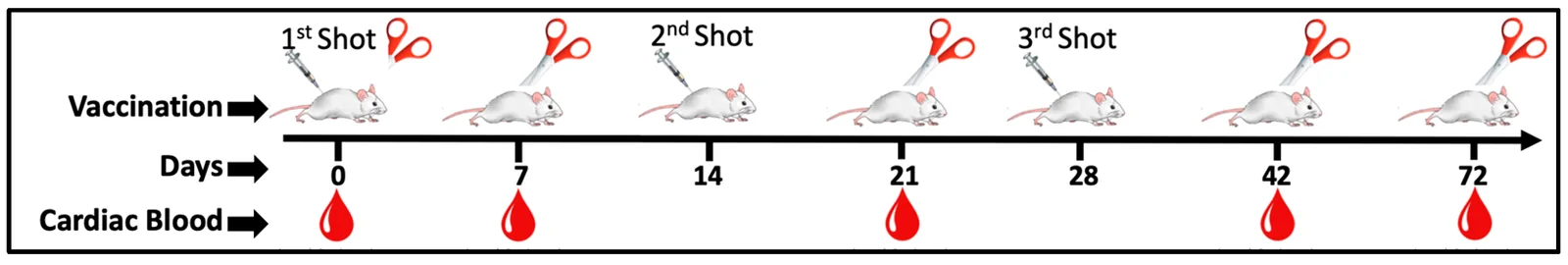
Experimental design for 14-day vaccination interval.

The 14-day vaccination interval was used, where the prime dose was given on day 0, and the first booster dose on day 14, the second booster dose on day 28 and the animals were monitored up to day 72 (Figure 1). At five time points (i.e., day 0, 7, 21, 42 and 72), a mouse was randomly picked from each cage and humanely euthanised by chloroform inhalation for approximately 30 s using chloroform-soaked cotton wool, after which cardiac blood samples were collected (Figure 1).

The mice were housed at the Makerere University, Uganda, Central Laboratory Animal Research Facility (CLARF) under standard husbandry conditions in polypropylene cages containing appropriate bedding material and environmental enrichment. Animals had ad libitum access to standard rodent feed and clean drinking water and were maintained under controlled environmental conditions with a 12 h light/dark cycle, ambient temperature of 22 ± 3 °C, and relative humidity of 40–70%. Animals were monitored daily for general health and welfare, including appearance, behaviour, mobility, food and water intake, and signs of pain, distress, or adverse reactions following vaccination. Humane endpoints were predefined in accordance with the approved animal ethics protocol and included severe or persistent clinical signs, marked reduction in mobility, inability to access food or water, or substantial body weight loss. Any animal meeting the humane endpoint criteria would be humanely euthanised to minimise suffering.

#### 2.2.2. Determination of the Effects of the Vaccine Candidate on the Organ Functions

The effects of the vaccine candidate on the organ functions were determined on some selected organs (i.e., liver, kidney, heart, brain, and skeletal muscles). This was determined by conducting a biochemistry test on the serum obtained from the mice at the five time-points (i.e., day 0, 7, 21, 42 and 72). The serum is obtained from cardiac blood, which is collected from the euthanised mice through the cardiac puncture technique, into the microtube containing a clot activator. A panel of 11 parameters were used and these include (Albumin (ALB), Total protein (TP), Total bilirubin (TBIL), Direct Bilirubin (DBiL), Aspartate aminotransferase (AST), Alanine aminotransferase (ALT), Alkaline phosphatase (ALP), Gamma-glutamyl transferase (GGT), Urea (UREA), creatinine (CREA), and creatine kinase (CK). The chemistry analysis was performed using the Zybio EXC200 chemistry analyser.

#### 2.2.3. Determination of the Effects of the Vaccine Candidate on the Blood Components

The effects of the products on the blood components were determined by conducting haematology tests on the whole cardiac blood samples obtained from the mice at the five time-points (i.e., day 0, 7, 21, 42 and 72). About 200 µL of whole blood was collected in a microtube containing dipotassium ethylenediaminetetraacetic acid (K_2_EDTA), which acts as an anticoagulant by chelating calcium to maintain sample stability for haematology testing. The whole blood samples were analysed using Zybio Z5 Vet, a 5-part differential (5-diff) haematology analyser tailored for veterinary practices, offering 29 parameters, scattergrams, and histograms for comprehensive blood cell analysis. It has species-specific algorithms to provide accurate, complete blood count (CBC) results for various animals, including mice. In this study, our report prioritised the following blood components: white blood cells, red blood cells, platelets, and haemoglobin concentration.

#### 2.2.4. Determination of the Effects of the Vaccine Candidate on the Body Temperatures of the Mice

A change in the body temperature of mice after receiving a vaccine product is an indicator of systemic toxicity of the product. Therefore, the body temperatures of the mice were measured using a calibrated temperature gun every day the three vaccine shots were given (i.e., on day 0 (prime dose), day 14 (first booster) and day 28 (second booster)) before vaccination and 1 h after vaccination.

#### 2.2.5. Determination of the Effects of the Vaccine Candidate on the Body Weights of the Mice

To screen for the general toxicity of vaccines, the body weight of vaccine-treated animals was analysed as a general safety test [24,25]. The effects of the vaccine candidate on the body weights of the mice were determined by weighing the mice both at baseline (start of the experiment) and after every 7 days (i.e., Day 0, 7, 14, 21, 28 and follow-up on Day 42 and 72).

#### 2.2.6. Statistical Analysis

The statistical analysis was performed using GraphPad Prism 8.0.2. The normal distribution of the data was determined using the Shapiro–Wilk test. Then the mean differences between the test groups (i.e., mice that received vaccine at low, moderate and high doses, and empty CAV-2 vector) and the control group (i.e., mice that received the physiological buffer) were determined using either the One-way ANOVA, while the mean differences between the body temperatures taken before vaccination and after vaccination were determined using either the paired *t*-test since the data sets were found to be normally distributed. A *p*-value of 0.05 was considered. For the hematological and biochemical analyses, data from the five sampling time points were pooled within each treatment group, resulting in an effective sample size of 15 animals per group. Pooling the data increased the statistical power of the analyses while still allowing timely detection of any adverse effects that could arise during the course of the study. Physiological reference ranges were established from measurements obtained in the physiological buffer control group and were used as study-specific reference intervals for interpretation of safety outcomes. This approach was adopted to account for potential variability associated with animal source and local husbandry conditions.

## 3. Results

### 3.1. Liver Function Remains Unaltered Following Vaccination

**Figure 2:**
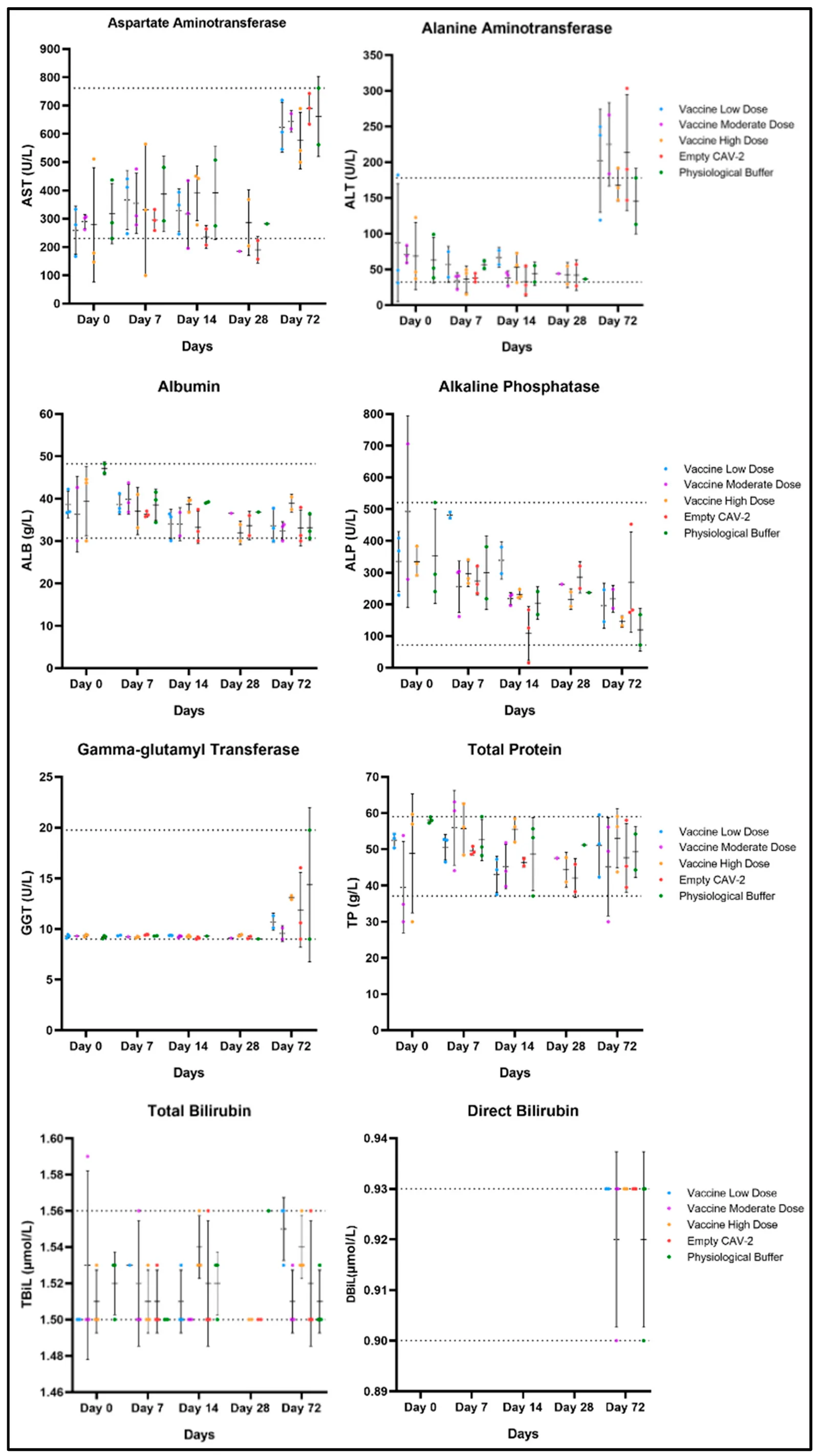
The effects of the vaccine candidate on liver functions. One-Way ANOVA test showed that at a *p*-value of 0.05, there were no statistically significant differences between the test groups (mice that received the vaccine and empty vector) and the control group (mice that received the physiological buffer (*n* = 15, *p* > 0.05)): AST (*p* = 0.9794), ALT (*p* = 0.6664), ALB (*p* = 0.2908), ALP (*p* = 0.1978), TP (*p* = 0.3171), GGT (*p* = 0.7657), TBiL (*p* = 0.841), and DBiL (*p* = 0.4411).

No evidence of hepatocellular injury or impaired hepatic function was observed across all vaccine dose groups, and in comparison of the test groups with the control group, there were no significant differences. Serum samples from a total of 15 mice (*n* = 15) were analysed for each parameter. Alanine aminotransferase (ALT) (*p* = 0.6664) and aspartate aminotransferase (AST) (*p* = 0.9794) levels remained within normal physiological ranges (dashed lines) throughout the study period, with only transient fluctuations observed at later time points (notably Day 72), which were comparable to those seen in control groups and not consistently associated with increasing vaccine dose. Markers of biliary function, including alkaline phosphatase (ALP) (*p* = 0.1978), gamma-glutamyl transferase (GGT) (*p* = 0.7657), total bilirubin (TBiL) (*p* = 0.841) and direct bilirubin (DBiL) (*n* = 3, *p* = 0.4411), showed no consistent elevations indicative of cholestasis or impaired bile excretion; bilirubin levels in particular remained stable across all groups and time points. Additionally, parameters reflecting hepatic synthetic function, including albumin (ALB) (*p* = 0.2908) and total protein (TP) (*p* = 0.3171), were maintained within normal ranges with no significant deviations between vaccinated and control animals (Table 2 and Figure 2).

### 3.2. Renal Function Remains Unaffected Following Vaccination

**Figure 3:**
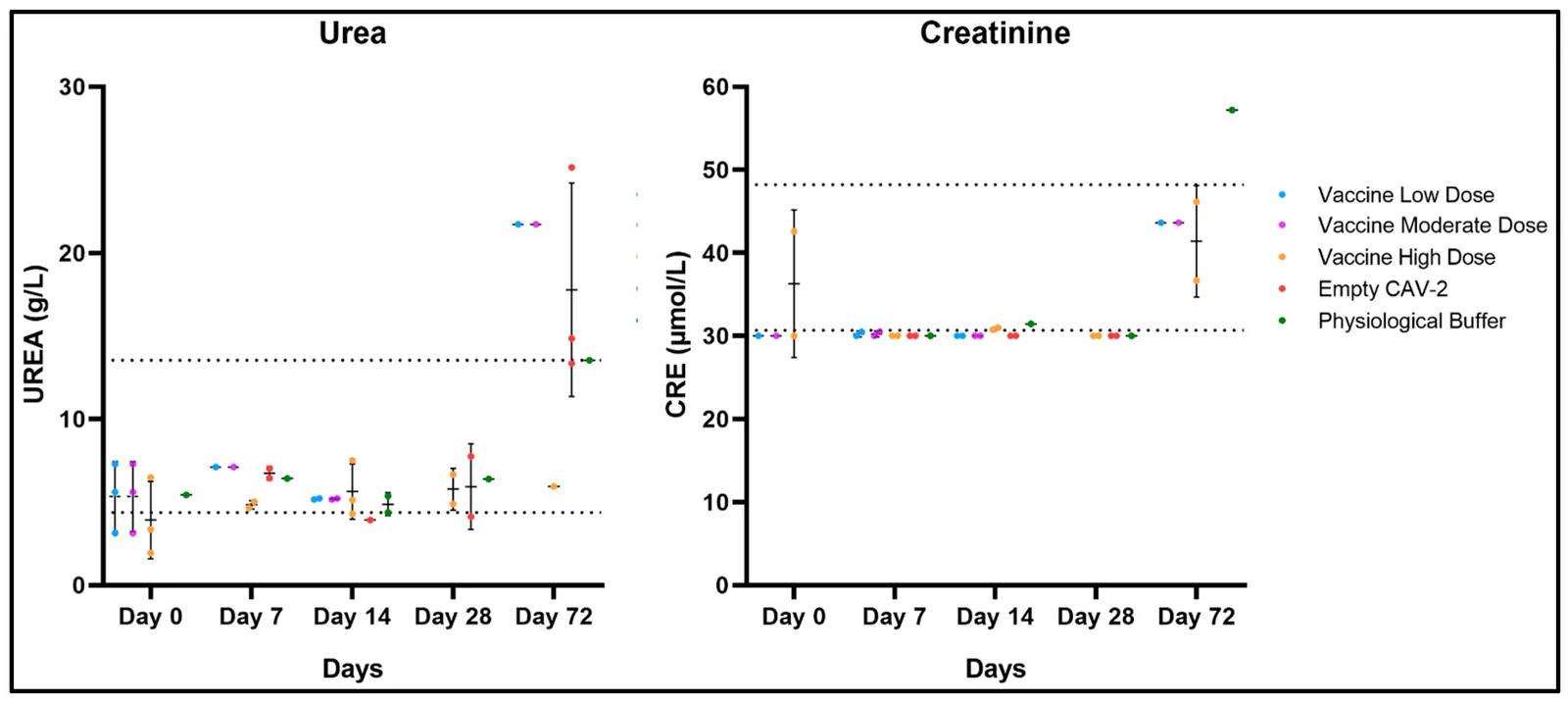
The effects of the vaccine candidate on kidney function. One-Way ANOVA test showed that at a *p*-value of 0.05, there were no statistically significant differences between the test groups (mice that received the vaccine and empty vector) and the control group (mice that received physiological buffer (*n* = 15, *p* > 0.05): urea (*p* = 0.242) and creatinine (*p* = 0.5313).

Vaccination did not induce detectable renal dysfunction or nephrotoxicity in mice. Serum urea (*n* = 15, *p* = 0.2420) and creatinine (*n* = 15, *p* = 0.5313) concentrations had no significant differences from the control group throughout the study period (Table 2), with the concentrations remaining within normal physiological ranges (dashed lines) across all vaccinated mice (Figure 3). These indicate that no overt nephrotoxicity was associated with the CAV-2 vector-based vaccine. Urea levels exhibited minor fluctuations across time points, with a transient increase observed at the terminal time point (Day 72); however, this elevation was not consistently associated with increasing vaccine dose and was similarly present in control groups. Creatinine concentrations remained stable and tightly clustered around baseline values (~30 µmol/L) for most of the study duration, with a slight increase noted at the final time point in some groups, again without a clear dose–response relationship. Importantly, these variations remained within acceptable reference ranges and were not indicative of compromised glomerular filtration (Figure 3).

### 3.3. No Evidence of Tissue Injury Following Vaccination

**Figure 4:**
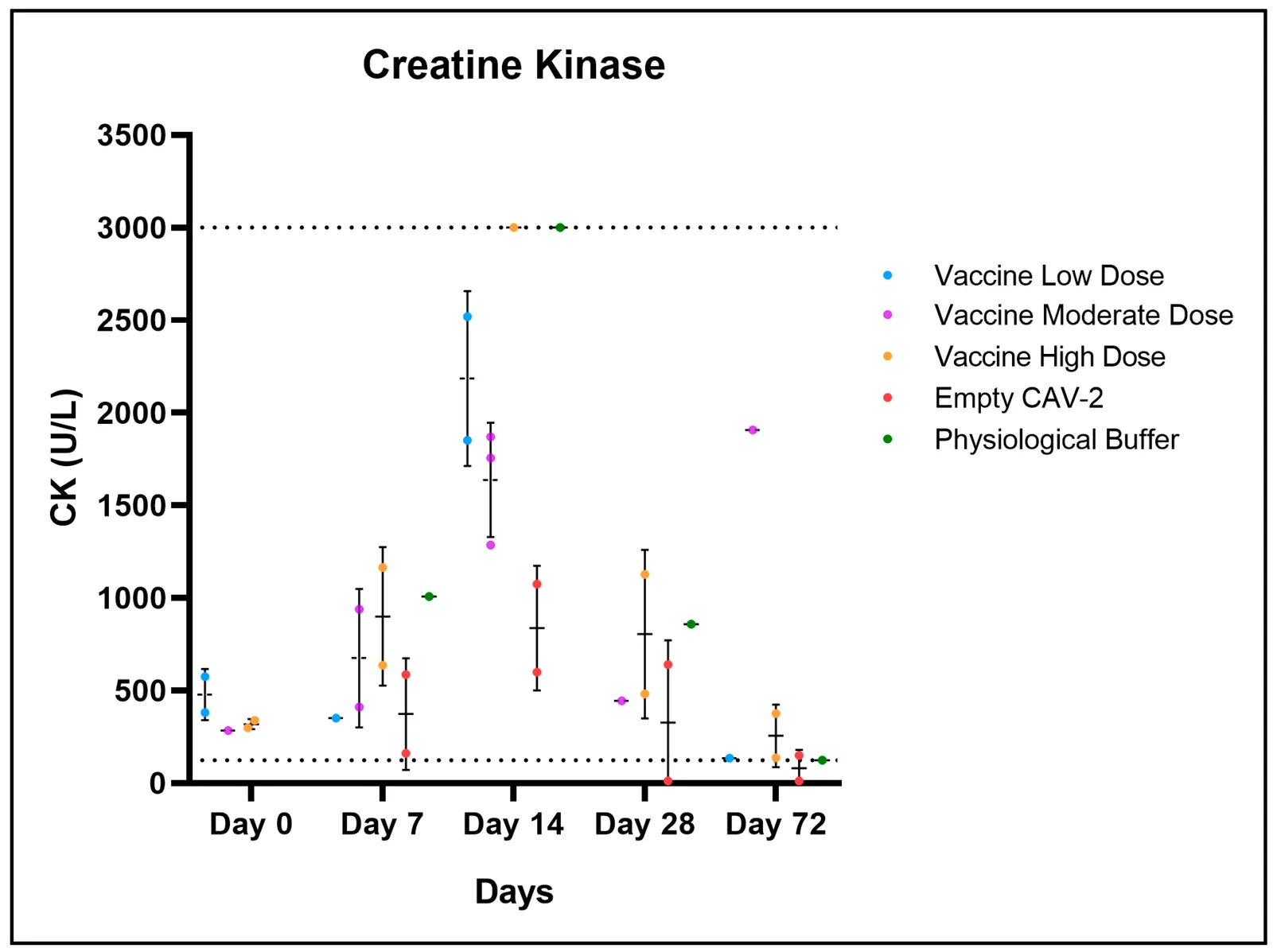
The effects of the vaccine candidate on serum creatine kinase as an indicator of tissue injury. One-Way ANOVA test showed that at a *p*-value of 0.05, there were no statistically significant differences in the serum creatine kinase concentrations between the test groups (mice that received the vaccine and empty vector) and the control group (mice that received physiological buffer (*n* = 15, *p* = 0.2981).

No evidence of sustained tissue injury was observed following vaccination. Total serum creatine kinase (CK) levels showed a transient increase following administration of the vaccine, with peak elevations observed at Day 14 across all vaccinated groups. These increases were variable and not consistently associated with increasing vaccine dose, and similar elevations were also noted in control groups with no significant differences (*n* = 15, *p* = 0.2981) (Table 2), suggesting a non-specific response, likely related to intramuscular injection or procedural factors. By Day 28, CK levels had declined substantially, and by Day 72, values had returned to near-baseline levels across all groups (Figure 4). Furthermore, no clinical signs indicative of neurological impairment, such as altered behaviour, locomotor deficits, or distress, were observed in any of the vaccinated groups throughout the study period. These findings indicate that administration of the CAV-2 vector-based vaccine has no measurable adverse effects on either the skeletal muscles, cardiac muscles, or neurological function in the murine model.

### 3.4. Haematological Parameters Remain Within Physiological Ranges Following Vaccination

**Figure 5:**
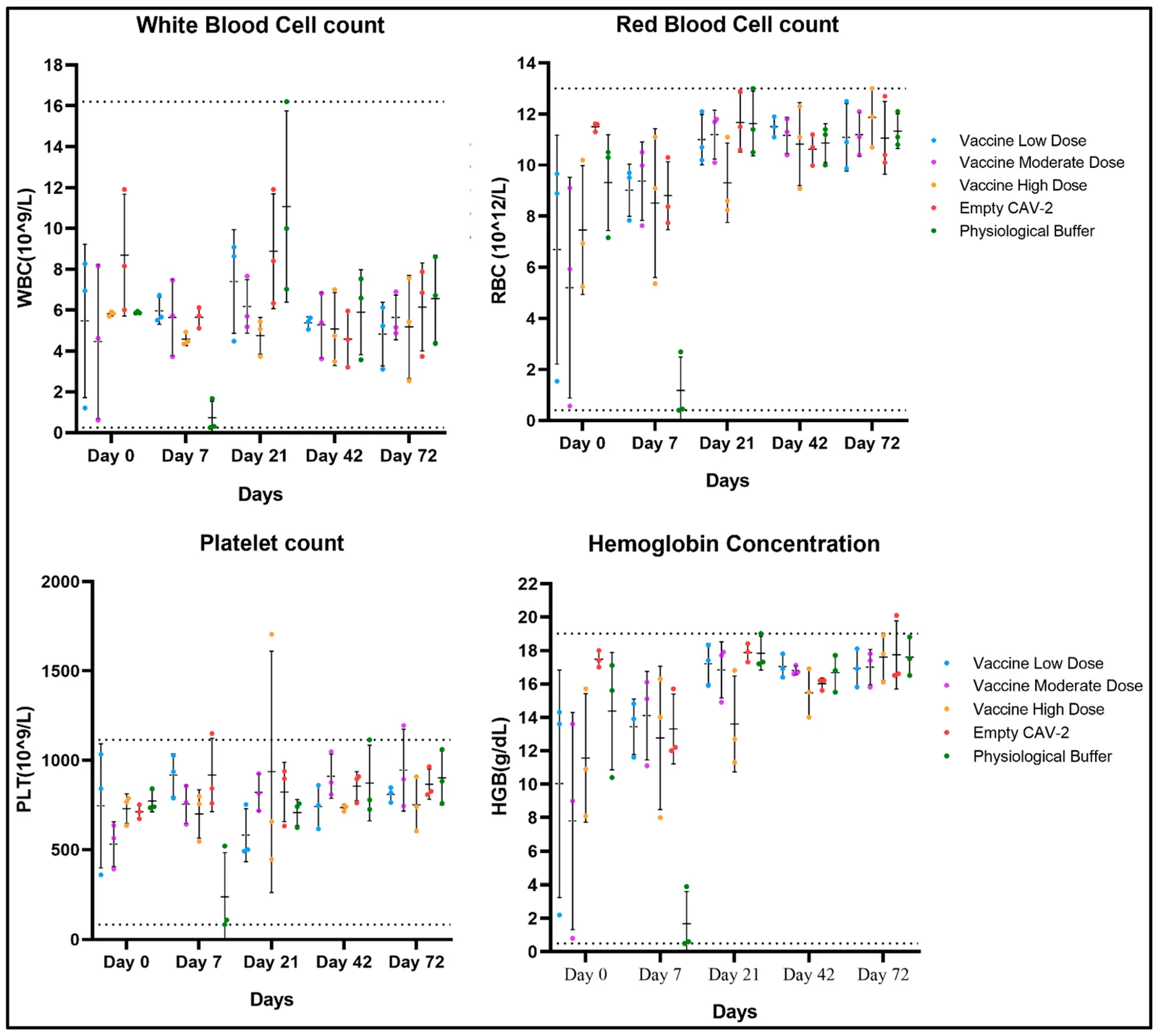
The effects of the vaccine candidate on the blood components. One-Way ANOVA test showed that at a *p*-value of 0.05, there were no statistically significant differences in the blood components between the test groups (mice that received the vaccine and empty vector) and the control group (mice that received physiological buffer (*n* = 15, *p* > 0.05)): WBC (*p* = 0.1199), RBC (*p* = 0.5446), PLT (*p* = 0.7601), and HGB (*p* = 0.3955).

The vaccine did not disrupt haematological homeostasis across any dose group. Haematological analysis of whole blood collected at Days 0, 7, 21, 42, and 72 showed that all measured parameters, including white blood cell (WBC) count, red blood cell (RBC) count, platelet count and haemoglobin (HGB) concentration, remained within normal physiological ranges (dashed lines) across vaccinated and control groups with no significant differences between the groups (*n* = 15): WBC (*p* = 0.1199), RBC (*p* = 0.5446), PLT (*p* = 0.7601), and HGB (*p* = 0.3955) (Table 2). Minor fluctuations were observed over time; however, these were not dose-related and were comparable between groups. No evidence of leukocytosis, anaemia, or thrombocytopenia was detected (Figure 5).

### 3.5. Body Temperature Remains Stable Following Vaccination

**Figure 6:**
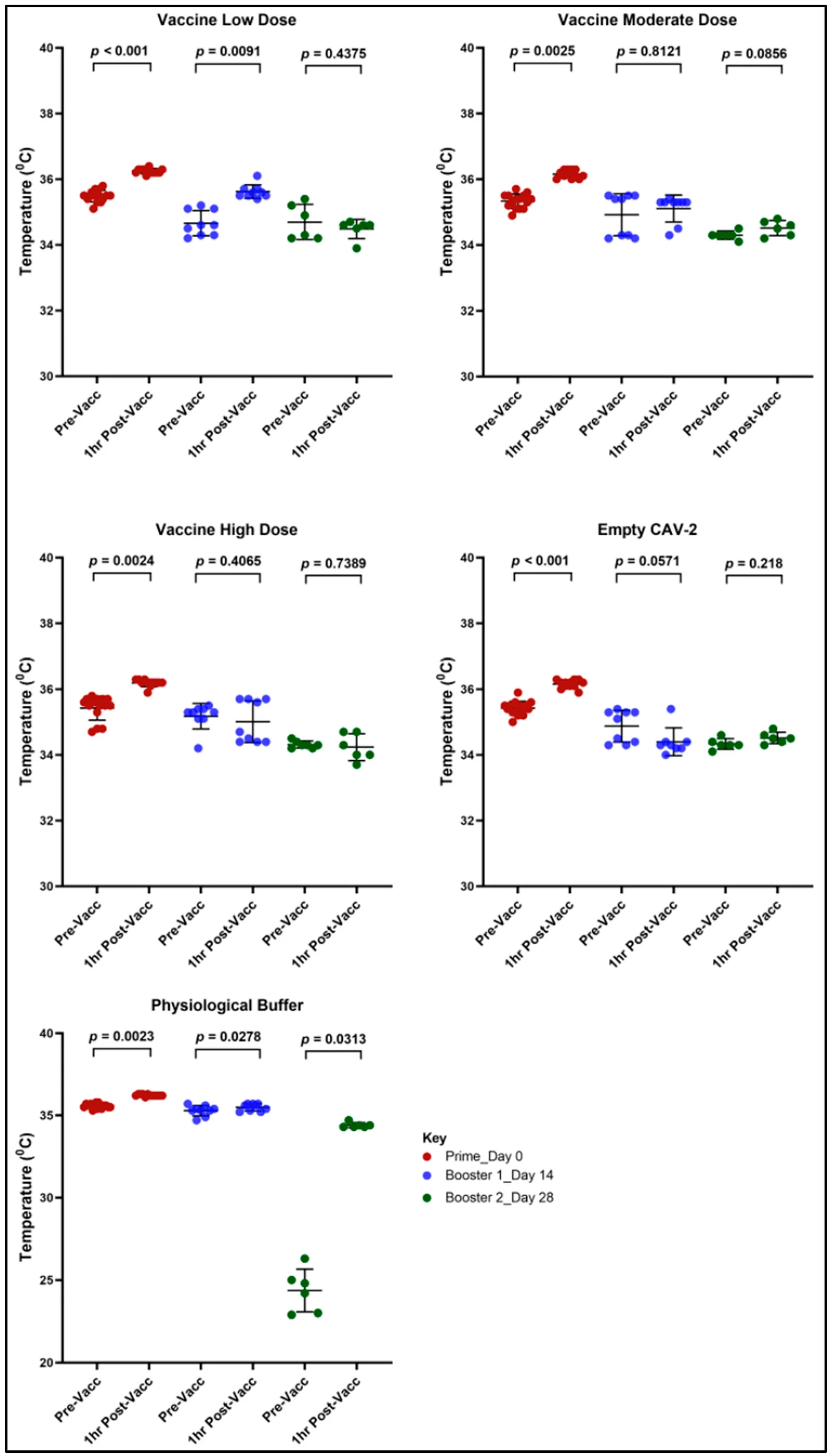
The effects of the vaccine candidate on the body temperatures of the mice. Generally, at a *p*-value of 0.05, the paired *t*-test showed that there were no statistically significant differences in the mice’s body temperatures before and after vaccination at the prime (*n* = 12), the first booster (*n* = 9) and the second booster (*n* = 6) time points, except in some selected cases where there was mild fever post-vaccination, with their *p*-values ranging from *p* = 0.0001 to *p* = 0.0091.

Vaccination did not induce clinically significant systemic toxicity or sustained thermoregulatory changes. Body temperature measurements taken before and one hour after administration of the prime (Day 0) and booster doses (Days 14 and 28) showed no evidence of clinically significant systemic toxicity associated with the CAV-2 vector-based vaccine. Across all dose groups (low, moderate, and high), only minor fluctuations in temperature were observed post-vaccination, with some statistically significant differences at specific time points (Table 2 and Figure 6); however, these changes were small in magnitude, transient, and not dose-related. Similar patterns were also observed in the control groups (physiological buffer and empty CAV-2 vector), indicating that the variations were likely attributable to handling or procedural factors rather than the vaccine itself. Importantly, no sustained febrile responses or hypothermic events were recorded following any of the three vaccine administrations (Figure 6).

### 3.6. Body Weight Remains Stable Following Vaccination

**Figure 7:**
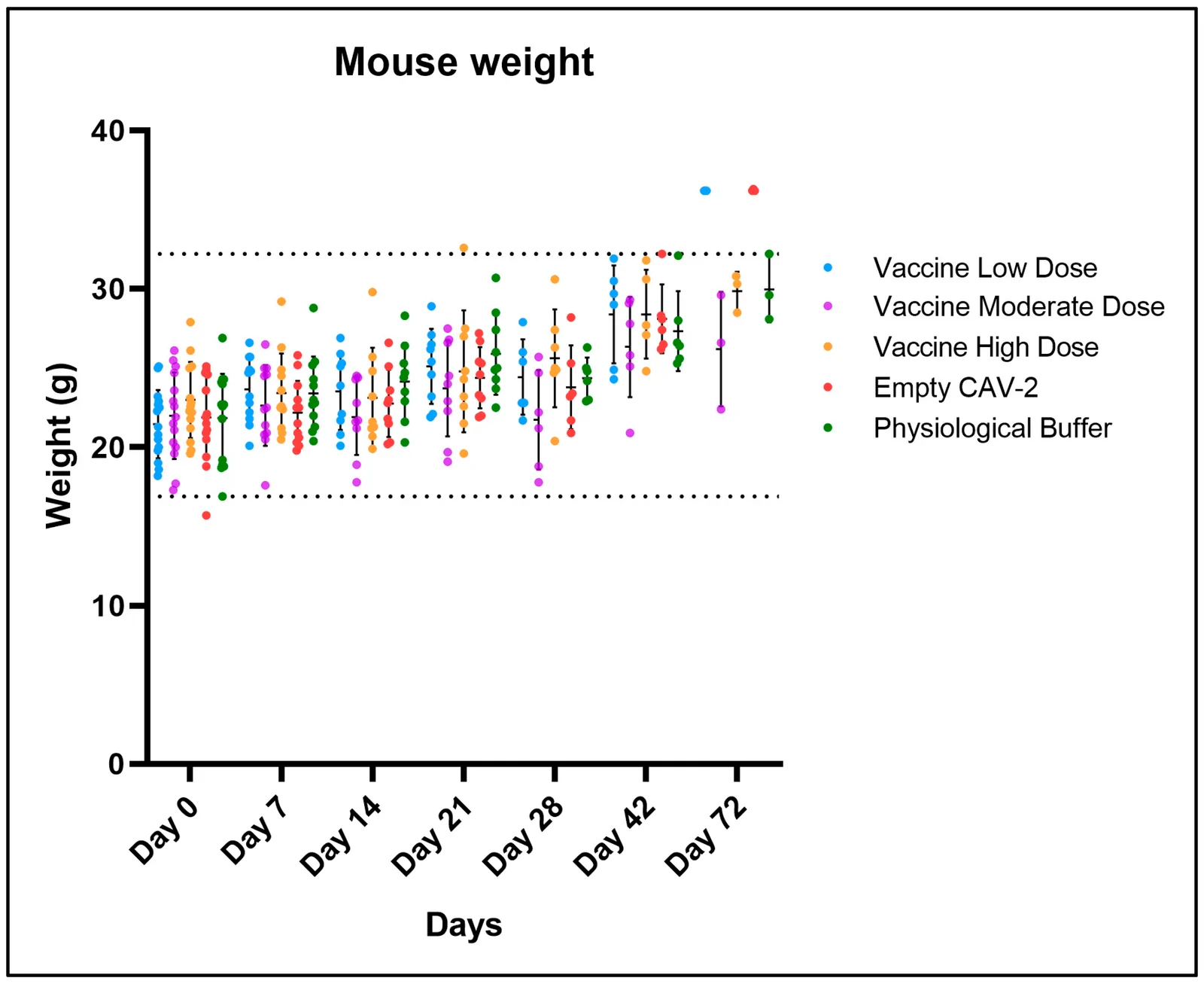
The effects of the vaccine candidate on the body weights of the mice. One-way ANOVA test showed that at a *p*-value of 0.05, there were no statistically significant differences in the mice’s body weights the test groups (mice that received the vaccine and empty vector) and the control group (mice that received physiological buffer) on Day 0 (*n* = 15, *p* = 0.3985), Day 7 (*n* = 12, *p* = 0.3627), Day 14 (*n* = 9, *p* = 0.5834), Day 21 (*n* = 9, *p* = 0.7618), Day 28 (*n* = 6, *p* = 0.1334), Day 42 (*n* = 6, *p* = 0.5528), and Day 72 (*n* = 3, *p* = 0.0004).

Normal growth trajectories were maintained throughout the study period, indicating the absence of systemic toxicity. Longitudinal assessment of body weight from day 0 to day 72 demonstrated a steady increase across all groups, consistent with normal growth patterns in BALB/c mice. One-way ANOVA test showed no significant growth difference between the test groups (i.e., mice that received vaccine and empty vector) and the control group (i.e., mice that received physiological buffer) on Day 0 (*n* = 15, *p* = 0.3985), Day 7 (*n* = 12, *p* = 0.3627), Day 14 (*n* = 9, *p* = 0.5834), Day 21 (*n* = 9, *p* = 0.7618), Day 28 (*n* = 6, *p* = 0.1334), and Day 42 (*n* = 6, *p* = 0.5528) (Table 2 and Figure 7). Except on Day 72 (*n* = 3, *p* = 0.0004), where there was significant mean differences in the mice body weights between the test groups ranging from 26.2 ± 3.6 g to 36.2 ± 0.1 g and control group 30.0 ± 2.1 g, most of the mice body weights were generally still within the normal body weight range of 16.9 g to 32.2 g, with the exception of some outliers (Table 2 and Figure 7). No significant weight loss or growth retardation was observed in any of the vaccinated groups (low, moderate, or high dose) compared to controls (empty CAV-2 vector and physiological buffer). Minor fluctuations occurred at intermediate time points; however, these changes were not consistently associated with increasing vaccine dose and fell within expected physiological variability (dashed lines). By the later time points (Days 42 and 72), mice in all groups exhibited comparable weight gain trajectories, with some individuals exceeding baseline ranges, further indicating good overall health status. Importantly, no sustained decrease in body weight, a key indicator of systemic toxicity, was observed following any of the vaccine administrations (Figure 7).

## 4. Discussion

This study provides a comprehensive preclinical evaluation of the safety profile of a replication-defective canine adenovirus type 2 (CAV-2) vector-based vaccine candidate in a murine model. Using a longitudinal design, multiple indicators of systemic toxicity were assessed, including serum biochemical parameters, haematological indices, and body temperature and weight changes. Such an integrated approach is consistent with recommended frameworks for preclinical vaccine safety assessment, which emphasise the use of complementary physiological, biochemical, and histopathological endpoints to detect potential adverse effects prior to clinical translation [26]. Given the increasing interest in alternative adenoviral vectors to overcome limitations associated with pre-existing immunity to human adenoviruses, establishing the safety profile of the CAV-2 vector is particularly relevant [27]. The findings presented herein are discussed in the context of current knowledge on adenoviral vector biology and vaccine safety, with emphasis on the implications for further development and translational application of CAV-2 vector-based vaccine platforms.

The absence of clinically significant changes in liver function biomarkers following administration of the CAV-2 vector–based vaccine indicates a favourable hepatic safety profile consistent with expectations for replication-defective adenoviral vectors. Serum transaminases (ALT and AST), which are sensitive indicators of hepatocellular injury, remained within physiological ranges with only transient, non-dose-related variations, suggesting the absence of sustained hepatocyte damage; elevations in these enzymes are typically associated with membrane leakage following hepatocellular necrosis or inflammation [28]. Similarly, stable levels of ALP, GGT, and bilirubin indicate preserved biliary function and lack of cholestasis, as perturbations in these markers are commonly linked to bile duct obstruction or impaired bile flow [29]. The maintenance of albumin and total protein concentrations further supports intact hepatic synthetic capacity, which is typically compromised only in cases of significant or chronic liver dysfunction [30]. Importantly, the observed biochemical stability across all dose groups aligns with previous reports demonstrating that non-human adenoviral vectors, including CAV-2 vector, exhibit low hepatotoxicity due to reduced off-target liver tropism compared to some human adenovirus serotypes [26]. Collectively, these findings suggest that the CAV-2 vector does not induce hepatocellular injury, cholestasis, or impairment of synthetic function in vivo, supporting its suitability as a safe vaccine delivery platform.

The stability of serum urea and creatinine levels across all groups indicates that the CAV-2 vector-based vaccine candidate did not demonstrate evidence of nephrotoxicity in the murine model. Urea and creatinine are widely accepted markers of renal function, reflecting nitrogen metabolism and glomerular filtration rate (GFR), respectively; sustained elevations typically indicate impaired renal clearance or kidney injury [31]. In this study, both parameters remained within physiological ranges, with only minor, non-dose-related fluctuations observed, including a transient increase in urea at the terminal time point that was also present in control groups. Such variations are commonly attributed to non-specific factors, including hydration status, protein metabolism, or procedural stress, rather than intrinsic renal dysfunction [32]. The absence of sustained creatinine elevation is particularly important, as creatinine is a more specific indicator of reduced GFR and kidney injury [33]. Furthermore, these findings are consistent with previous studies demonstrating that replication-defective adenoviral vectors generally exhibit minimal renal toxicity when administered intramuscularly, owing to limited systemic dissemination and low renal tropism [34]. Hence, the biochemical stability observed supports the conclusion that the vaccine candidate does not impair renal function, reinforcing its favourable safety profile.

The transient elevation in total serum creatine kinase (CK) observed post-vaccination, peaking around Day 14 and returning to near-baseline by Day 72, is consistent with a short-lived, non-specific response rather than sustained tissue injury. CK is a sensitive but non-specific biomarker released following muscle membrane disruption, including that induced by intramuscular injections or physical handling, and transient increases are commonly reported in such contexts without indicating pathological damage [35]. The absence of a clear dose–response relationship and the presence of similar CK elevations in control groups further support a procedural rather than vaccine-specific effect. Importantly, persistent or progressive CK elevation, typically associated with myopathy or cardiac injury, was not observed [36]. In addition, no clinical signs of neurological dysfunction, such as altered behaviour or impaired locomotion, were detected, suggesting the absence of neurotoxicity. These findings align with previous studies indicating that replication-defective adenoviral vector-based vaccines administered intramuscularly have minimal off-target toxicity and limited systemic distribution [34]. These clinical data support the conclusion that the CAV-2 vector-based vaccine candidate did not demonstrate evidence of muscle, cardiac, or neurological toxicity in this preclinical model. However, a limitation of this study is that only total serum creatine kinase (CK) was measured; therefore, the findings could not distinguish between skeletal muscle, cardiac muscle, or neurological tissue injury.

The maintenance of haematological parameters within normal physiological ranges across all time points indicates that the CAV-2 vector-based vaccine does not adversely affect hematopoiesis or peripheral blood homeostasis. Key indices, including white blood cell (WBC) count, red blood cell (RBC) count, haemoglobin concentration, and platelet count, remained stable, with only minor fluctuations observed that were not consistently associated with increasing vaccine dose, suggesting the absence of systemic inflammation, bone marrow suppression, or haematological toxicity. WBC counts are sensitive indicators of immune activation or infection, while RBC and haemoglobin levels reflect erythropoietic function, and platelet counts are critical for assessing coagulation status; significant deviations in these parameters are typically associated with pathological processes such as infection, anaemia, or thrombocytopenia [37]. The lack of such deviations in this study supports normal physiological function. Furthermore, the absence of haematological abnormalities is consistent with previous reports demonstrating that replication-defective adenoviral vectors exhibit minimal haematological toxicity in preclinical models [26]. Therefore, these findings reinforce the favourable safety profile of the vaccine candidate, with no evidence of adverse effects on blood cell production or function.

The absence of sustained changes in body temperature following administration of the prime and booster doses indicates that the CAV-2–based vaccine candidate does not elicit clinically significant systemic toxicity. Body temperature is a sensitive physiological marker of systemic inflammatory responses, with fever typically arising from cytokine-mediated pyrogenic pathways activated during infection or immune stimulation [38]. In this study, only minor, transient fluctuations were observed post-vaccination, with no consistent dose-related trends and similar variations present in control groups, suggesting that these changes are more likely attributable to handling stress or procedural factors rather than vaccine-induced effects. This is consistent with reports that mild, short-lived temperature variations can occur in laboratory animals due to environmental or experimental handling conditions or a mild side-effect that is common in vaccine response [39]. Importantly, the absence of sustained febrile or hypothermic responses across all vaccination time points supports the conclusion that the vaccine does not trigger systemic inflammatory toxicity. These findings are in line with previous studies demonstrating that replication-defective adenoviral vector-based vaccines administered intramuscularly are generally well tolerated and do not induce significant systemic adverse effects [40]. However, body temperature was assessed only before and 1 h after vaccination; delayed pyrogenic responses that may occur several hours or days following vaccine administration were not evaluated.

The consistent increase in body weight observed across all groups throughout the study period indicates that the CAV-2 vector-based vaccine did not demonstrate evidence of systemic toxicity or adversely affect general health status in the murine model. Body weight is a sensitive and widely used indicator of overall safety in preclinical studies, with sustained weight loss or impaired weight gain often reflecting underlying toxicity, metabolic disruption, or disease processes [41]. In this study, all groups, including vaccinated and controls, exhibited normal growth trajectories typical of BALB/c mice, with no evidence of dose-related effects or growth retardation. Minor fluctuations observed at intermediate time points were within expected physiological variability and likely attributable to normal biological variation or handling stress. The absence of weight loss following repeated vaccine administration further supports good tolerability, as adverse systemic reactions are frequently associated with reduced food intake and subsequent weight decline [42]. These findings indicate that replication-defective adenoviral vector-based vaccines administered intramuscularly are generally well tolerated and do not negatively impact growth or metabolic health. Thus, the maintenance of normal weight gain patterns and the stability of body temperature reinforce the favourable safety and tolerability profile of the vaccine candidate.

In this study, the safety assessment of the replication-defective CAV-2-vectored SARS-CoV-2 vaccine candidate focused primarily on clinical, physiological, haematological, and biochemical endpoints. It did not include additional histopathological and toxicological evaluations such as biodistribution, vector persistence, and vector shedding. These would provide a more comprehensive understanding of the long-term safety of the vaccine following administration. Although the study employed multiple vaccine dose levels, appropriate control groups, and longitudinal monitoring, the sample size at each terminal sampling time point was limited, which may have reduced the statistical power to detect subtle biological differences. In addition, the study was conducted exclusively in BALB/c mice, and therefore, the findings may not be fully generalisable to other animal models or humans. Therefore, further studies involving larger cohorts and evaluation in additional animal models will be necessary to support the continued translational development of CAV-2 as an alternative adenoviral vaccine vector.

## 5. Conclusions

This study contributes preclinical evidence that the replication-defective canine adenovirus type 2 (CAV-2) vector-based vaccine candidate exhibits a favourable safety profile in BALB/c mice. These findings support the translational potential of CAV-2 as an alternative adenoviral vector-based vaccine platform. Future studies should focus on comprehensive immunogenicity profiling to correlate safety with protective efficacy, as well as biodistribution and persistence studies to better understand vector kinetics in vivo. Evaluation in higher-order animal models, including non-human primates, is recommended to support translational relevance and regulatory advancement. Additionally, dose optimisation and long-term safety assessments, including repeat-dose toxicity, local injection-site and organ histopathology evaluations, will be critical for progression toward clinical trials.

## Figures and Tables

**Table 1:** The blinded study design.

Product	Test Group	Colour Code	Cage No	Sex	Cage No	Sex	Cage No	Sex	Sub-Total Females	Sub-Total Males	Total
Vaccine	Low Dose (1 × 10^6^ PFU)	Red	10	5 F	9	5 M	1	5 M	5 F	10 M	15 Mice
	Moderate Dose (0.5 × 10^10^ PFU)	Dark-Green	12	5 F	6	5 F	5	5 M	10 F	5 M	15 Mice
	High Dose (1 × 10^10^ PFU)	Light-Yellow	22	5 F	11	5 M	21	5 M	5 F	10 M	15 Mice
Control	Empty Vector (0.5 × 10^10^ PFU)	Blue	18	5 F	17	5 M	13	5 M	5 F	10 M	15 Mice
	Physiological Buffer	Orange	20	5 F	7	5 M	19	5 M	5 F	10 M	15 Mice
									30	45	75

**Table 2:** Group sample sizes for the parameters measured, test statistics used and their *p*-values, means, and their standard deviations over the 72-day study period.

Parameter	Physiological Buffer	Vaccine Low Dose	Vaccine Moderate Dose	Vaccine High Dose	Empty CAV-2	*n*-Value	One-Way ANOVA Test Statistic	*p*-Value
Chemistry analysis								
	Mean ± SD	Mean ± SD	Mean ± SD	Mean ± SD	Mean ± SD			
AST (U/L)	425.3 ± 173.4	400.1 ± 169.2	372.3 ± 159.9	396.5 ± 172.1	380.5 ± 220.02	15	0.06	0.9794
ALT (U/L)	68.7 ± 47.5	103.7 ± 83.5	78.5 ± 76.7	78.8 ± 57.5	85.1 ± 90.8	15	0.53	0.6664
ALB (g/L)	37.8 ± 5.0	35.7 ± 3.4	36.4 ± 4.5	38.2 ± 4.5	34.1 ± 3.1	15	1.29	0.2908
ALP (U/L)	224.2 ± 86.6	333.7 ± 123.8	282.3 ± 155.9	248.4 ± 71.4	229.4 ± 114.9	15	1.63	0.1978
GGT (U/L)	10.5 ± 3.7	9.6 ± 0.7	9.3 ± 0.4	9.9 ± 1.5	10.0 ± 2.2	15	0.38	0.7657
TP (g/L)	51.8 ± 6.6	48.9 ± 6.1	47.9 ± 10.0	53.7 ± 6.5	46.9 ± 5.6	15	1.21	0.3171
TBiL (µmol/L)	1.5 ± 0.02	1.5 ± 0.03	1.5 ± 0.05	1.5 ± 0.02	1.5 ± 0.03	15	0.28	0.8410
DBiL (µmol/L)	0.9 ± 0.00	0.9 ± 0.00	0.9 ± 0.02	0.0 ± 0.00	0.9 ± 0.00	15	1.00	0.4411
UREA (g/L)	6.9 ± 3.3	8.3 ± 6.8	7.9 ± 6.3	5.1 ± 1.6	10.3 ± 7.2	15	1.47	0.2420
CREA (µmol/L)	37.2 ± 13.4	32.4 ± 5.5	32.4 ± 5.5	33.7 ± 6.0	39.0 ± 16.8	15	0.75	0.5313
CK (U/L)	1247.6 ± 1230.6	969.4 ± 975.34	1112.4 ± 687.05	840.2 ± 885.7	405.0 ± 380.0	15	1.29	0.2981
Hematology analysis								
	Mean ± SD	Mean ± SD	Mean ± SD	Mean ± SD	Mean ± SD			
WBC (10^9^/L)	6.0 ± 4.0	5.7 ± 2.1	5.4 ± 1.9	5.1 ± 1.3	6.8 ± 2.5	15	2.03	0.1199
RBC (10^12^/L)	8.9 ± 4.2	9.9 ± 2.7	9.6 ± 3.0	9.6 ± 2.4	10.7 ± 1.4	15	0.72	0.5446
PLT (10^9^/L)	698.4 ± 285.5	753.2 ± 199.7	792.2 ± 195.6	770.4 ± 281.9	835.1 ± 131.1	15	0.39	0.7601
HGB (g/dL)	13.6 ± 6.4	15.0 ± 4.1	14.5 ± 4.6	14.2 ± 3.4	16.5 ± 2.1	15	1.16	0.3955
Body Weight (g)								
	Mean ± SD	Mean ± SD	Mean ± SD	Mean ± SD	Mean ± SD			
Day 0	21.8 ± 2.8	21.5 ± 2.2	22.0 ± 2.7	23.0 ± 2.4	21.9 ± 2.6	15	1.00	0.3985
Day 7	23.4 ± 2.3	23.7 ± 2.0	22.6 ± 2.5	23.4 ± 2.5	22.2 ± 2.0	12	1.09	0.3627
Day 14	24.2 ± 2.4	23.5 ± 2.4	21.9 ± 2.4	23.1 ± 3.2	22.8 ± 2.1	9	0.66	0.5834
Day 21	25.9 ± 2.6	25.1 ± 2.4	23.7 ± 3.0	24.8 ± 3.8	24.4 ± 1.9	9	0.39	0.7618
Day 28	24.4 ± 1.3	24.4 ± 2.4	21.7 ± 3.1	25.6 ± 3.1	23.8 ± 2.6	6	2.08	0.1334
Day 42	27.3 ± 2.5	28.4 ± 3.1	26.3 ± 3.2	28.4 ± 2.8	28.1 ± 2.2	6	0.72	0.5528
Day 72	30.0 ± 2.1	36.2 ± 0.0	26.2 ± 3.6	29.9 ± 1.2	36.2 ± 0.1	3	20.26	0.0004
Body Temperature (°C): Paired *T*-test								
	Mean ± SD	Mean ± SD	Mean ± SD	Mean ± SD	Mean ± SD			
Prime-Pre-Vaccination	35.5 ± 0.2	35.3 ± 0.2	35.4 ± 0.4	35.4 ± 0.2	35.6 ± 0.1			
Prime-1hr Post-Vaccination	36.3 ± 0.1	36.2 ± 0.1	36.2 ± 0.1	36.2 ± 0.1	36.2 ± 0.1			
	(*n* = 12, *p* = 0.00)	(*n* = 12, *p* < 0.05)	(*n* = 12, *p* = 0.0025)	(*n* = 12, *p* = 0.0024)	(*n* = 12, *p* < 0.5)			
Booster1-Pre-Vaccination	34.7 ± 0.4	34.9 ± 0.6	35.2 ± 0.4	34.9 ± 0.5	35.3 ± 0.3			
Booster1-1hr Post-Vaccination	35.6 ± 0.2	35.1 ± 0.4	35.0 ± 0.6	34.4 ± 0.4	35.5 ± 0.2			
	(*n* = 9, *p* = 0.0278)	(*n* = 9, *p* = 0.0091)	(*n* = 9, *p* = 0.8121)	(*n* = 9, *p* = 0.4065)	(*n* = 9, *p* = 0.0571)			
Booster2-Pre-Vaccination	34.7 ± 0.5	34.3 ± 0.1	34.3 ± 0.1	34.3 ± 0.2	24.4 ± 1.3			
Booster2-1hr Post-Vaccination	34.5 ± 0.3	34.5 ± 0.2	34.2 ± 0.4	34.5 ± 0.2	34.4 ± 0.2			
	(*n* = 6, *p* = 0.0313)	(*n* = 6, *p* = 0.4375)	(*n* = 6, *p* = 0.0856)	(*n* = 6, *p* = 0.7389)	(*n* = 6, *p* = 0.218)			

## Data Availability

All relevant data are within the manuscript.
